# Mosaic loss of chromosome Y in peripheral blood cells is associated with age-related macular degeneration in men

**DOI:** 10.1186/s13578-022-00811-9

**Published:** 2022-05-31

**Authors:** Qinchun Duan, Yuru Gao, Xixi Cao, Shulin Wang, MengMeng Xu, Odell D. Jones, Xuehong Xu

**Affiliations:** 1 Laboratory of Cell Biology, Genetics and Developmental Biology, Shaanxi Normal University College of Life Sciences, and University Hospital Medical Center, West Chang’an 620, District Chang’an, Xi’an, 710119 People’s Republic of China; 2 Department of Pediatrics, Morgan Stanley Children’s Hospital, Columbia University, 3959 Broadway, New York, NY 10032 USA; 3 University of Pennsylvania School of Medicine ULAR, Philadelphia, PA 19144 USA; 4 Shaanxi Normal University College of Life Sciences, University Hospital Medical Center, Xi’an, 710062 People’s Republic of China

**Keywords:** Age-related macular degeneration (AMD), Mosaic loss of Y chromosome (mLOY), Hematopoietic stem cells (hSC), Monocyte-macrophage differentiation system, Mitosis/meiosis

## Abstract

**Background:**

Age-related macular degeneration (AMD) is the leading cause of severe vision loss in patients over 55 years old in the industrialized world. In the past 20 years, approximately 288 million patents have been affected by this disease. Despite this high prevalence, the molecular mechanism for AMD remains unclear, and there remains no effective treatment for this disease. The mosaic loss of Y chromosome (mLOY) has been identified as a common phenomenon in multiple age-related disease (i.e., oncogenesis and cardiovascular disease) has recently been identified by genome-wide analysis to be linked to AMD as well. As the Y chromosome mainly possesses three genomic functions, sister chromatin cohesion, cell cycle mitosis, and apoptotic signaling, here we characterize the Y chromosome euchromatic genes and non-chromosome AMD genes in relevance to cellular proliferation and apoptotic signaling of leukocytes.

**Results:**

Using STRING, a publically available database of all protein–protein interaction, Grassmann et al. found the genes on the Y chromosome is mainly believed to take part in three major cellular genomic functions- sister chromatin cohesion, cell cycle mitosis, and apoptotic signaling. Based on data from the Ensembl Genome database, we focus on our discussion on coding genes found in the euchromatins but not the PAR1 and PAR2 regions of the Y chromosomes. All 14 known euchromatic genes on the Y chromosome short arm and all 31 known euchromatic genes on the Y chromosome long arm (Yq) are directly or indirectly involved in the cell cycle (meiosis and mitosis) and proliferation. We sorted non-Y chromosome AMD associated genes into these three categories to identify signaling pathways that may compound with cellular dysregulation due to mLOY. Of the genes associated with AMD, complement pathway genes such as *C2*,* C9* and *CFH/ARMD4* are associated with proliferation, receptor-mediated endocytosis genes such as *APOE*, *DAB2* and others associated with apoptotic signaling. Because nucleated cells found in peripheral circulation are mainly composed of leukocytes with reduced expression of CD99, a protein essential for leukocytes adhesion, translocation, and function, mLOY in these cells likely affect retinal degeneration through altered immunological surveillance. In fact, there is precedence that circulating macrophage can stabilize and modify the cardiac rhythm and contractility post ischemic damage. Therefore, the most likely mechanism through which peripheral mLOY affects AMD development in men is through the role affected leukocytes play in retinal proliferation and apoptosis.

**Conclusions:**

mLOY in peripheral blood is newly discovered in AMD by Grassmann et al. as it is a common phenomenon in oncogenesis and cardiac dysfunction. Here the recent data conclude the possible mechanism for the newly identified link between mLOY and AMD, and provide support that mLOY in circulating macrophage-monocyte of affected male patients promotes AMD by targeting the retina and causing macular degeneration.

**Supplementary Information:**

The online version contains supplementary material available at 10.1186/s13578-022-00811-9.

As the leading cause of blindness, age-related macular degeneration (AMD) is responsible for severe vision loss and 10% of legal blindness in patients over age 55. Worldwide, AMD accounts for vision loss in eight million patients. In 20 years, this population is expected to reach 288 million globally [1]. In the United States (US), AMD associated vision impairment is projected to double by 2050. The incidence of AMD is also rising in developing countries. Given this global rise in disease prevalence and burden, early diagnosis and treatment of AMD would be beneficial to a large number of patients. Understanding the molecular mechanisms contributing to AMD pathology is essential to identifying those at risk, early disease, and potential treatments.

Grading of AMD as early, intermediate, or advanced disease, and geographic and neovascular AMD is dependent on a variety of factors. These include lesion location, size, and number and also cellular. Advanced AMD is then further characterized by the presence or absence of retinal pigment epithelial detachment, serous or hemorrhagic retinal detachment, subretinal pigmentation, epithelial hemorrhage, subretinal fibrous tissue, hard exudates, and photocoagulation scars. Retinal pigment epithelial abnormalities and drusen morphology are also used to characterize severe AMD [1]. Although multiple mitochondrial proteins have been associated with genetic susceptibility to AMD, these markers have not been used to identify patient risk or further elucidate the pathophysiological mechanisms in the disease. A recent report on decreased Y-chromosome stability in patients with high risk of age-related macular degeneration provides another potential promising avenue for molecular biology based early clinical diagnosis and prognosis in AMD [2–4]. Here we discuss these findings and the results of experiments exploring the molecular mechanisms behind the importance of the Y-chromosome in AMD.

Y-specific single-nucleotide polymorphisms (SNPs) have been used to demonstrate a precedence of strong correlation between mosaic loss of Y chromosome (mLOY) and a variety of other age-related diseases, such as neurodegenerative diseases, oncologic processes, and cardiac dysfunction. However, a joint publication between biostatisticians and geneticists at the Karolinska institute, University of Regensburg, University of Paris, and the International Age-related Macular Degeneration Genomics Consortium (IAMDGC) led by Veitia, was the first to report the mLOY in nucleated blood cells to be strongly associated with late-stage AMD in 5772 male patients with AMD controlled against 6732 male patients without AMD [2]. Analysis of a dataset independent from this first study population confirmed cells patients with AMD to have a higher fraction of mLOY cells. Furthermore, based on population studies, Y chromosome loss seemed to present a selective advantage over XY cells and male patients with AMD seem to have a predisposition for mLOY earlier in life than control subjects. Although they were not characterize mLOY of the same patient as the patient ages, the Veitia group confirmed by population samples that the prevalence of mLOY increased significantly with age. And confirmed that independent of age, risk for advanced AMD was strongly associated with mLOY in patients between the ages of 65 and 75 [2].

In a study separate from the genome-wide population study described above, the same group subsequently analyzed molecular cytogenetics and genotyping microarray data to further confirm a significant correlation between the fraction of mLOY and AMD [3]. This analysis was based on well-documented data amassed over a half-a-century’s worth of peripheral blood cells samples from patients. Using protein–protein interaction data on previous published datasets, Grassmann et al. performed Gene-set enrichment analysis on a large population of AMD patients that further confirmed patients with AMD to have a mean frequency of mLOY significantly larger than controls from ages 50 to 100 years [2, 5]. Their data further demonstrated that patients in the AMD cohort seem to have a greater tendency to loss their Y chromosome at a younger age [3]. Their analysis put the AMD cohort at three times more likely to have mLOY than the non-AMD risk population. Considering in humans only approximately 5% of peripheral blood cells contain DNA, this strong association between peripheral mLOY and AMD must be largely due to mLOY induced changes in leukocytes activity.

Using STRING, a publically available database of all protein–protein interaction [5], Grassmann et al. systematically analyzed the genomic function of Y-chromosome based on protein–protein interactions to further identify the key functions of this chromosome. Their data derived from 85,542 male individuals from 19 genomic regions, characterized genes found on the Y-chromosome into three categories including sister chromatin cohesion (*p* = 0.004), cell cycle mitosis (*p* = 0.003) and apoptotic signaling (*p* = 0.001). Genes *PMF1* on 1q22, *MAD1L1* (7p22.3) and *CENPN* (16q23.2) implicated linking function of Y-chromosome euchromatin genes to sister chromatid cohesion and cell cycle/mitosis, while *TPX2* (20q11.21) and *TP53* (17p13.1) were implicated linking function of Y-chromosome euchromatin genes to the apoptotic signaling pathway [2, 5]. From their findings, they inferred mLOY to result in cellular abnormality in circulating leukocytes, leading to targeting and destruction of the retina by these immune cells, thereby leading to macular degeneration in men. Apoptotic signaling pathway could cause geographic atrophy as characterized by the loss of photoreceptors and retinal pigment epithelium. Abnormality in sister chromatid cohesion and cell cycle/mitosis can be associated with superfluous growth of choriocapillaries which develops into neovascular AMD, which is characterized by extreme neovascularization of the choroid within Bruch’s membrane (Fig. 1).

**Fig. 1:**
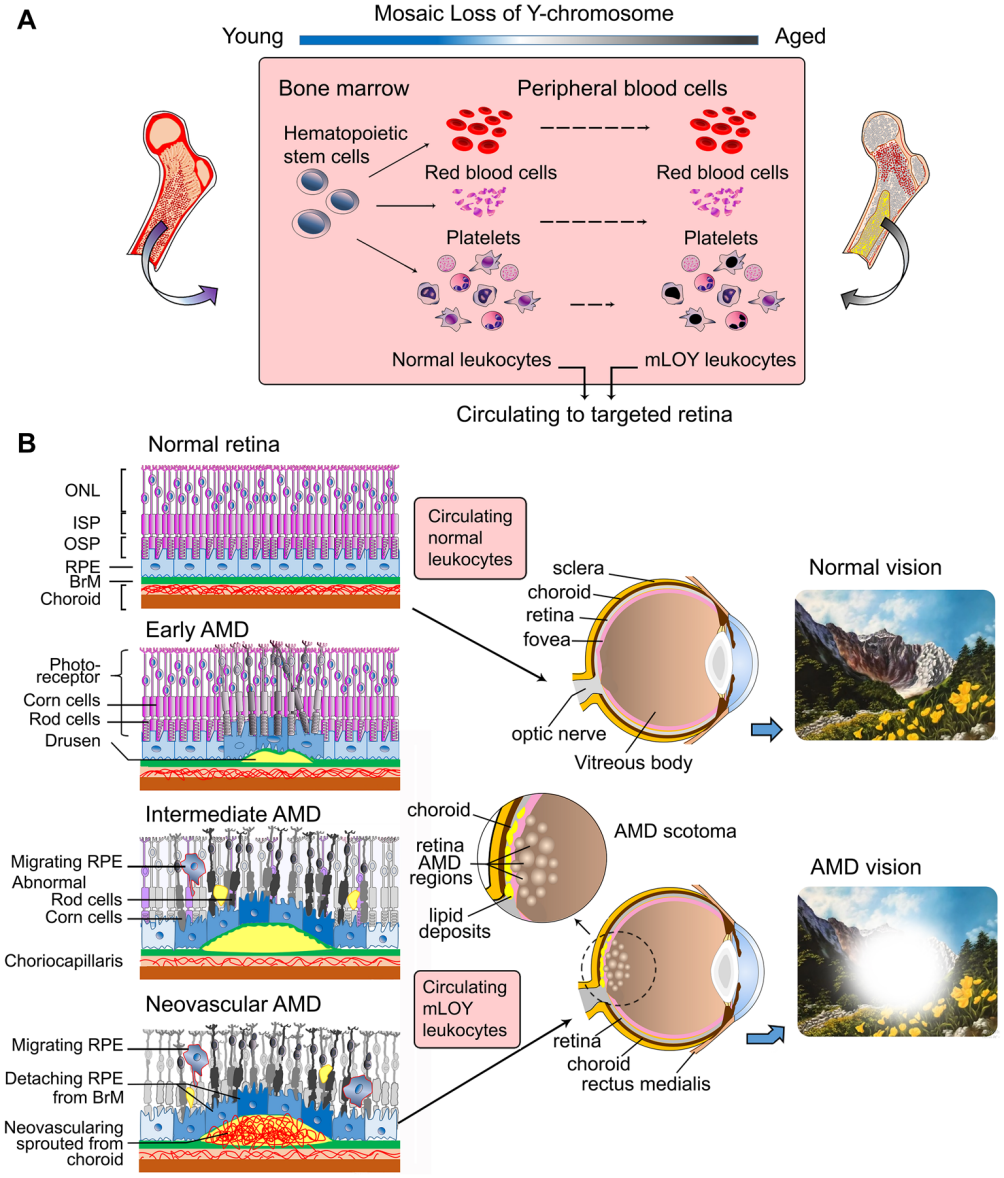
Age-related macular degeneration (AMD) and mosaic Loss of Y-chromosome (mLOY) in ageing. **A** Development of monocyte-macrophage system associated mLOY from bone marrow to peripheral blood; **B** Circulating mLOY leukocyte targeting on retina leads to AMD vision. ONL, outer nuclear layer; ISP, inner segment, photoreceptor; OSP, outer segment, photoreceptor; RPE, retinal pigment epithelium; BrM, Bruch’s membrane

Sister chromatin cohesion, cell cycle mitosis and apoptotic signaling are basic functions of the Y-chromosome as described by Grassmann et al. According to Ensembl Genome Browser data, the ~ 57 million base pairs (mb) human Y genome contains 64 coding and 109 non-coding genes in addition to 395 pseudogenes. The PAR1 region is found on the distal end of the Y-chromosome short arm (Yp) and contains 15 genes including *ASMT*, *SHOX*, and *CD99* while the PAR2 region found on the long arm (Yq) of the Y-chromosome contains two genes *VAMP7* and *IL9R*. X-chromosome also contains these PAR1 and PAR2 regions. For our purposes, we will limit our discussion to only the 14 coding genes found on Yp, which make up 15.12% of the Yp euchromatin genome, and the 31 coding genes found on Yq, which make up 10.22% of the Yq euchromatin genome.

Amongst these 14 known genes on Yp, five genes are directly associated with cell cycle and proliferation, while the other nine genes are indirectly involved in these cellular processes (Fig. 2 and Additional file 1: Table S1). For instance, the seven testes-specific protein on Y chromosome (TSPY) members including TSPY1, TSPY2, TSPY3, TSPY4, TSPY8, TSPY9P and TSPY10 have been shown to play a critical role in renewal of spermatogonial stem cells and meiotic divisions by inhibiting Cyclin Dependent Kinase 1 (CDK1-cyclin B) phosphorylation. The two zinc finger protein Y-linked (ZFY) found on Yp are likewise indirectly associated with promoting meiotic division and regulation of apoptotic signaling.

**Fig. 2:**
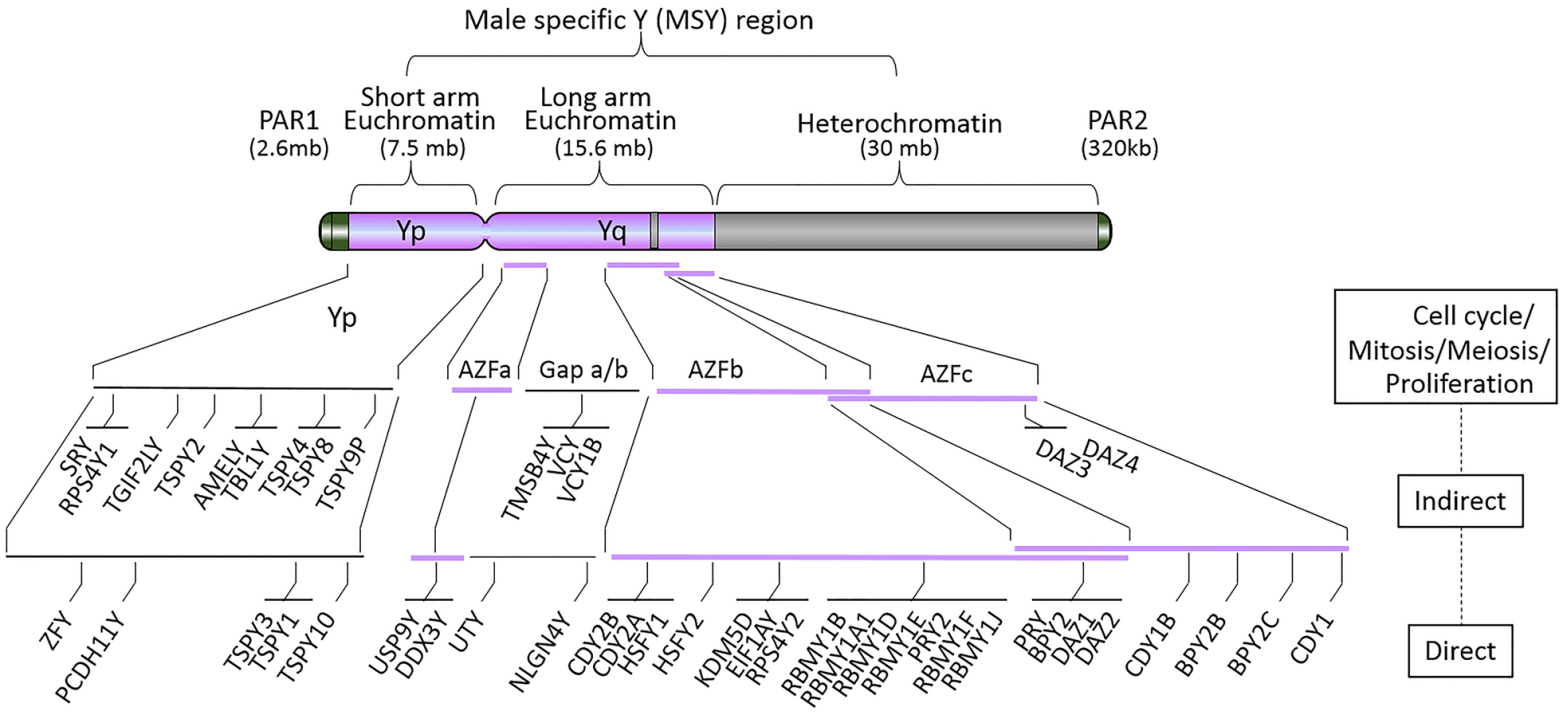
Structure of the human Y chromosome with male specific region (MSY) and the PAR1 and PAR2 regions labeled. The pseudo autosomal regions (PAR1 and PAR2) are located at two terminals of the Y chromosome. The short arm of the human Y chromosome (Yp) contains 14 genes, which are all directly or indirectly-related to cell cycle/mitosis/meiosis/proliferation. The long arm of the human Y chromosome (Yq) is composed of euchromatin encoding for azoospermia factors (AZFa, AZFb and AZFc) and with a gap between AZFa and AZFb (Gap a/b). There is also a genetically inactive heterochromatin region distributed between AZFc and PAR2

Amongst the 31 known genes on Yq, 26 genes are directly associated with cell cycle and proliferation while 5 genes are indirectly involved in these cellular processes (Fig. 2 and Additional file 1: Table S1). The three Azoospermia Factor (AZF) loci AZFa, AZFb, and AZFc distributed in Yq are responsible for meiotic arrest. Mutations in these three genes lead to the absence of post-meiotic germ cells and are causes of male infertility due to cryptozoospermia and oligozoospermia. For example, an increasing number of reports found large deletions in the Yq11 region of AZFb (Yq11.1–11.23) result in spermatogenic disruptions during or after meiosis during human male germ cell development [6]. Large deletions in the P5/proximal-P1.2 region of Yq along with microdeletions in this area can also affect other genes critical to meiotic arrest by disrupting members of the *RBMY*, *BPY*, *CDY*, and *DAZ* gene families.

In total, of the 45 known Y chromosome genes 11.81% of the Y euchromatic genome is involved in aspects of the cell cycle/meiosis/proliferation. Five genes on the Y chromosome are also functionally linked to apoptotic signaling including ZFY, lysine demethylase 5D (KDM5D), RNA binding motif protein Y-linked family 1 member B (RBMY1B) and PTPN13 like Y-linked genes (PRY2 and PRY).

To survey whether defects in functions in these three large categories might contribute to AMD, we studied these proposed functions of genes found on the Y chromosome (sister chromatin cohesion, mitosis, and apoptosis) in association with known AMD functions which not distribute on the Y chromosome. According to the recent reviews on AMD-related protein coding genes and non-coding microRNA [1, 2], 76 of the 124 genes found on the Y-chromosome fall directly into these two categories. Of coding genes associated with AMD, 19 and 11 (of 49) are functionally linked to cellular proliferation and apoptotic signaling, respectively. Seven of these genes fall into both categories (Additional file 2: Table S2). Of the known AMD related pathways, the complement pathway is highly implicated, with C2 (or ARMD14), C9 (or ARMD15) and factor H (or CFH/ARMD4) well known to be involved in cellular proliferation. Receptor-mediated endocytosis associated with apolipoprotein E, DAB adaptor protein 2, heat shock protein family H (Hsp110) member 1 and MICAL like 1 are also likely involved in apoptotic signaling. In addition, collagen type VIII alpha 1 chain and integrin subunit alpha 7 are marker proteins of endodermal cell differentiation, with lipid pathway apolipoprotein E (APOE) also associated with proliferation. As an important protein in positive regulation of neuroblast proliferation, spindle microtubules assembly factors (ASPM) are critically involved in both proliferation and apoptotic signaling along with ARMD3 and VEGFA only associated to proliferation (Additional file 2: Table S2). Although ARMD1/Fibulin-6 was the first gene suspected in AMD based on genetic analysis of a large family with predisposition towards AMD, conflicting reports since its identification has challenged this finding. Regardless of this controversy, multiple in vitro knockout, RNA interference, and parthenogenesis experiments show that deletion of this gene leads directly to mitotic arrest during embryonic cell division. Deletion of this gene leads to arrest of cytokinesis and cleavage furrow formation between sister cells. With the subsequent mitotic arrest resulting in multi-nucleated cells that then succumb to apoptotic nuclear disruptions.

mLOY has been associated with multiple other age-related diseases which provide some insight into how mLOY could cause disease [7]. Leukocytes with 45 or X0 karyotype dominate in leukemic or pre-leukemic patients’ bone marrow and mortality rates are strongly associated with mLOY [2]. mLOY is associated with the development and increase in mortality in a variety of other oncologic diseases, such as colorectal, bladder, lung, and androgen associated cancers (i.e., prostate and testicular germ cell tumors) [8]. Outside of the oncologic field, mLOY has also been implicated in hypertension and cardiovascular disease. Genetics studies of patients and murine animal models have both linked mLOY in lymphocytes and macrophages to immunodeficiency and autoimmune pathology. Based on these findings, mLOY in circulating cells could reflect a decreased fitness in hematopoietic stem cells, which then generate abnormally functioning in lymphocytes and macrophages that fail to correct or facilitate pathogenesis and disease progression in various organs [2, 3].

Recent papers published by Forsberg et al. provided a wider window of understanding for the molecular and cellular mechanism of mLOY effect on disease phenotype, which can stream comprehensive understanding to AMD as well. This paper strongly linked mLOY in leukocytes to increased mortality and morbidity in male patients across multiple disease processes. Using genomic DNA and RNA analysis of sorted- and single-cell leukocytes in vivo and in vitro, this group identified a LOY-associated transcriptional effect (LATE) that affected the expression of over 500 genes in male Alzheimer’s disease patients with mosaic Y chromosome loss. Furthermore, RNA variation data correlated this LATE with the rate of mLOY in both individual patients and affected cell types [9]. Importantly, Forsberg’s group discovered that mosaic LOY is accompanied by reduction of CD99 immunoprotein abundance on the membrane surface of leukocytes [10]. Notably, the *CD99* gene is the last gene located on the pseudoautosomal region-1 (PAR1) adjacent to the sex determining region Y (*SRY* gene) on the short arm of the Y-chromosome. Interestingly, the *CD99* gene is also found on the short arm of the X-chromosome, located at the boundary the X-chromosome PAR1 region adjacent to Xg glycoprotein (*XG*) gene. However, *CD99* escapes X-inactivation in females, thus balanced expression of this gene is especially delicate in male patients. The *CD99* gene is likely to play a key role in the immune protection against disease processes as surface expression of this glycoprotein is essential for cell-cell adhesion of immune cells. The presence of *CD99* facilitates transendothelial migration (TEM), a key step in immune cell localization, monitoring, and removal of diseased cells, including in those found in AMD (Additional file 3).

Known associations between mLOY in peripheral cells and disease in other organ systems provide some hints to how mosaic loss of the Y chromosome in circulating leukocytes might lead to AMD. It has been known for years that differentiation of circulating monocytes-macrophages is closely linked to recovery post myocardial infarction (MI) [11]. In cardiac studies, pluripotent monocyte-macrophage labelled with cell surface antigens CCR2/CD192, CD64 (also known as FcγR1), CX3CR1, and Mac3 effectively migrate to the cardiac muscle and participate in essential repair of damaged tissue. Recently, Hulsmans et al. provided direct evidence proving macrophages from peripheral blood can manipulate cardiac electrical conduction and promote cardiac regeneration [12]. Using GFP labelled macrophages, the experiments clearly showed that after the tissue-specific naturalization, the macrophages localize to and disburse at critical points of the conduction system to conduct post-ischemic repair and assist in electrical conduction. Macrophages distributed along the AV-His bundle were shown to have their cytoplasmic body extending between cardiomyocytes across long distances [12]. In these instances, macrophages were shown to bridge intercalated discs and help preserve muscular contraction. This hypothesis is supported by a comprehensive analysis of 141,533 samples of monocytes and macrophages from 918 human participants showing that loss of the Y chromosome in peripheral blood is associated with increased risk of coronary artery disease in men [13]. Therefore, we propose that peripheral monocytes and macrophages that have lost the Y chromosome may also facilitate AMD through altered immunity and inflammation leading to poor retinal repair or extra growth of Neovascular (proliferation), or increased retinal damage (apoptosis).

## Supplementary Information

**Additional file 1**: **Table S1.** Y-chromosome euchomatic genes associate to cell cycle meiosis/proliferation and apoptotic signaling.

**Additional file 2**: **Table S2.** AMD genes and Y-chromosome function categorized as the proliferation and apoptotic signaling.

**Additional file 3.** Additional references.

## Data Availability

Not applicable.

## Abbreviations

AMD: Age-related macular degeneration
APOE: Apolipoprotein E
ASPM: Spindle microtubules assembly factor
AZF: Azoospermia factor
Hsp: Heat shock protein family H
LATE: LOY-associated transcriptional effect
KDM5D: Lysine demethylase 5D
mLOY: Mosaic loss of Y-chromosome
MSY: Male specific region
PAR: Pseudo autosomal region
PRY: PTPN13 like Y-linked gene
RBMY1B: RNA binding motif protein Y-linked family 1 member B
SRY: Sex determining region Y
TSPY: Testis specific protein Y-linked
XG: Xg glycoprotein
ZFY: Zinc finger protein Y-linked
