# Supplementary material for: Mosaic loss of chromosome Y in peripheral blood cells is associated with age-related macular degeneration in men
Source: Cell Biosci. 2022 May 31;12:73. doi: 10.1186/s13578-022-00811-9 (PMC9153148; doi:10.1186/s13578-022-00811-9)
Supplement: Supplementary file 3 — Additional file 3. Additional references. [file 13578_2022_811_MOESM3_ESM.docx]

**Additional file 3. Additional references**

1. Taleahmad S, Alikhani M, Mollamohammadi S, Yousefi M, Taei A, Hassani SN, Baharvand H, Salekdeh GH. Inhibition of Human Y Chromosome Gene, SRY, Promotes Naïve State of Human Pluripotent Stem Cells. J Proteome Res. 2019;18(12):4254-61. https://doi.org/10.1021/acs.jproteome.9b00396.

2. Kaur G, Jans DA. Dual nuclear import mechanisms of sex determining factor SRY: intracellular Ca2+ as a switch. FASEB J. 2011;25(2):665-75. https://doi.org/10.1096/fj.10-173351.

3. Kiefer JC. Back to basics: Sox genes. Dev Dyn. 2007;236(8):2356-66. https://doi.org/10.1002/dvdy.21218.

4. Abdelalim EM, Emara MM, Kolatkar PR. The SOX transcription factors as key players in pluripotent stem cells. Stem Cells Dev. 2014;23(22):2687-99. https://doi.org/10.1089/scd.2014.0297.

5. Spena S, Cordiglieri C, Garagiola I, Peyvandi F. Development of a Specific Monoclonal Antibody to Detect Male Cells Expressing the RPS4Y1 Protein. Int J Mol Sci. 2021;22(4):2001. https://doi.org/10.3390/ijms22042001.

6. Sudhamalla B, Yadaiah M, Ramakrishna D, Bhuyan AK. Cysteine protease attribute of eukaryotic ribosomal protein S4. Biochim Biophys Acta. 2012;1820(10):1535-42. https://doi.org/10.1016/j.bbagen.2012.05.001.

7. Colaco S, Modi D. Genetics of the human Y chromosome and its association with male infertility. Reprod Biol Endocrinol. 2018;16(1):14. https://doi.org/10.1186/s12958-018-0330-5.

8. Affara NA, Chambers D, O'Brien J, Habeebu SS, Kalaitsidaki M, Bishop CE, Ferguson-Smith MA. Evidence for distinguishable transcripts of the putative testis determining gene (ZFY) and mapping of homologous cDNA sequences to chromosomes X,Y and 9. Nucleic Acids Res. 1989;17(8):2987-99. https://doi.org/10.1093/nar/17.8.2987.

9. Lau YF, Chan KM. The putative testis-determining factor and related genes are expressed as discrete-sized transcripts in adult gonadal and somatic tissues. Am J Hum Genet. 1989;45(6):942-52. PMID: 2511751.

10. Laity JH, Lee BM, Wright PE. Zinc finger proteins: new insights into structural and functional diversity. Curr Opin Struct Biol. 2001;11(1):39-46. https://doi.org/10.1016/s0959-440x(00)00167-6.

11. Aarabi M, Ousati-Ashtiani Z, Nazarian A, Modarressi MH, Heidari M. Association of TGIFLX/Y mRNA expression with azoospermia in infertile men. Mol Reprod Dev. 2008;75(12):1761-6. https://doi.org/10.1002/mrd.20906.

12. Dhanoa JK, Mukhopadhyay CS, Arora JS. Y-chromosomal genes affecting male fertility: A review. Vet World. 2016;9(7):783-91. https://doi.org/10.14202/vetworld.2016.783-791.

13. Anilkumar TR, Devi AN, Pillai SM, Jayakrishnan K, Oommen OV, Kumar PG. Expression of protocadherin 11Yb (PCDH11Yb) in seminal germ cells is correlated with fertility status in men. Reprod Fertil Dev. 2017;29(11):2100-11. https://doi.org/10.1071/RD16478.

14. Terry S, Queires L, Gil-Diez-de-Medina S, Chen MW, de la Taille A, Allory Y, Tran PL, Abbou CC, Buttyan R, Vacherot F. Protocadherin-PC promotes androgen-independent prostate cancer cell growth. Prostate. 2006;66(10):1100-13. https://doi.org/10.1002/pros.20446.

15. Yang X, Chen MW, Terry S, Vacherot F, Chopin DK, Bemis DL, Kitajewski J, Benson MC, Guo Y, Buttyan R. A human- and male-specific protocadherin that acts through the wnt signaling pathway to induce neuroendocrine transdifferentiation of prostate cancer cells. Cancer Res. 2005;65(12):5263-71. https://doi.org/10.1158/0008-5472.CAN-05-0162.

16. Vogel T, Schmidtke J. Structure and function of TSPY, the Y-chromosome gene coding for the "testis-specific protein". Cytogenet Cell Genet. 1998;80(1-4):209-13. https://doi.org/10.1159/000014982.

17. Lau YC, Li Y, Kido T. Battle of the sexes: contrasting roles of testis-specific protein Y-encoded (TSPY) and TSPX in human oncogenesis. Asian J Androl. 2019;21(3):260-9. https://doi.org/10.4103/aja.aja_43_18.

18. Jobling MA, Lo IC, Turner DJ, Bowden GR, Lee AC, Xue Y, Carvalho-Silva D, Hurles ME, Adams SM, Chang YM, et al. Structural variation on the short arm of the human Y chromosome: recurrent multigene deletions encompassing Amelogenin Y. Hum Mol Genet. 2007;16(3):307-16. https://doi.org/10.1093/hmg/ddl465.

19. Mitani K, Haruyama N, Hatakeyama J, Igarashi K. Amelogenin splice isoforms stimulate chondrogenic differentiation of ATDC5 cells. Oral Dis. 2013;19(2):169-79. https://doi.org/10.1111/j.1601-0825.2012.01967.x.

20. Meyfour A, Ansari H, Pahlavan S, Mirshahvaladi S, Rezaei-Tavirani M, Gourabi H, Baharvand H, Salekdeh GH. Y Chromosome Missing Protein, TBL1Y, May Play an Important Role in Cardiac Differentiation. J Proteome Res. 2017;16(12):4391-402. https://doi.org/10.1021/acs.jproteome.7b00391.

21. Tagariello A, Breuer C, Birkner Y, Schmidt S, Koch AM, Cesnjevar R, Ruffer A, Dittrich S, Schneider H, Winterpacht A, et al. Functional null mutations in the gonosomal homologue gene TBL1Y are associated with non-syndromic coarctation of the aorta. Curr Mol Med. 2012;12(2):199-205. https://doi.org/10.2174/156652412798889027.

22. Jain BP, Pandey S. WD40 Repeat Proteins: Signalling Scaffold with Diverse Functions. Protein J. 2018;37(5):391-406. https://doi.org/10.1007/s10930-018-9785-7.

23. Gegenschatz-Schmid K, Verkauskas G, Stadler MB, Hadziselimovic F. Genes located in Y-chromosomal regions important for male fertility show altered transcript levels in cryptorchidism and respond to curative hormone treatment. Basic Clin Androl. 2019;29:8. https://doi.org/10.1186/s12610-019-0089-3.

24. Navarro-Romero MT, Muñoz ML, Alcala-Castañeda E, Terreros-Espinosa E, Domínguez-de-la-Cruz E, García-Hernández N, Moreno-Galeana MÁ. A novel method of male sex identification of human ancient skeletal remains. Chromosome Res. 2020;28(3-4):277-91. https://doi.org/10.1007/s10577-020-09634-1.

25. Bhat MA, Sharma JB, Roy KK, Sengupta J, Ghosh D. Genomic evidence of Y chromosome microchimerism in the endometrium during endometriosis and in cases of infertility. Reprod Biol Endocrinol. 2019;17(1):22. <https://doi.org/10.1186/s12958-019-0465-z.>

26. Li Y, Zhang DJ, Qiu Y, Kido T, Lau YC. The Y-located proto-oncogene TSPY exacerbates and its X-homologue TSPX inhibits transactivation functions of androgen receptor and its constitutively active variants. Hum Mol Genet. 2017;26(5):901-12. <https://doi.org/10.1093/hmg/ddx005>.

27. Kido T, Lau YF. Roles of the Y chromosome genes in human cancers. Asian J Androl. 2015;17(3):373-80. https://doi.org/ 10.4103/1008-682X.150842.

28. Lee KH, Song GJ, Kang IS, Kim SW, Paick JS, Chung CH, Rhee K. Ubiquitin-specific protease activity of USP9Y, a male infertility gene on the Y chromosome. Reprod Fertil Dev. 2003;15(1-2):129-33. https://doi.org/10.1071/rd03002.

29. Vakilian H, Mirzaei M, Sharifi Tabar M, Pooyan P, Habibi Rezaee L, Parker L, Haynes PA, Gourabi H, Baharvand H, Salekdeh GH. DDX3Y, a Male-Specific Region of Y Chromosome Gene, May Modulate Neuronal Differentiation. J Proteome Res. 2015;14(9):3474-83. <https://doi.org/10.1021/acs.jproteome.5b00512.>

30. Gueler B, Sonne SB, Zimmer J, Hilscher B, Hilscher W, Græm N, Rajpert-De Meyts E, Vogt PH. AZFa protein DDX3Y is differentially expressed in human male germ cells during development and in testicular tumours: new evidence for phenotypic plasticity of germ cells. Hum Reprod. 2012;27(6):1547-55. <https://doi.org/10.1093/humrep/des047.>

31. Kotov AA, Olenkina OM, Godneeva BK, Adashev VE, Olenina LV. Progress in understanding the molecular functions of DDX3Y (DBY) in male germ cell development and maintenance. Biosci Trends. 2017;11(1):46-53. <https://doi.org/10.5582/bst.2016.01216.>

32. Ahn J, Kim KH, Park S, Ahn YH, Kim HY, Yoon H, Lee JH, Bang D, Lee DH. Target sequencing and CRISPR/Cas editing reveal simultaneous loss of UTX and UTY in urothelial bladder cancer. Oncotarget. 2016;7(39):63252-63260. <https://doi.org/10.18632/oncotarget.11207.>

33. Gažová I, Lengeling A, Summers KM. Lysine demethylases KDM6A and UTY: The X and Y of histone demethylation. Mol Genet Metab. 2019;127(1):31-44. <https://doi.org/10.1016/j.ymgme.2019.04.012.>

34. Warren EH, Gavin MA, Simpson E, Chandler P, Page DC, Disteche C, Stankey KA, Greenberg PD, Riddell SR. The human UTY gene encodes a novel HLA-B8-restricted H-Y antigen. J Immunol. 2000;164(5):2807-14. <https://doi.org/10.4049/jimmunol.164.5.2807.>

35. Wong HY, Wang GM, Croessmann S, Zabransky DJ, Chu D, Garay JP, Cidado J, Cochran RL, Beaver JA, Aggarwal A, et al. TMSB4Y is a candidate tumor suppressor on the Y chromosome and is deleted in male breast cancer. Oncotarget. 2015;6(42):44927-40. <https://doi.org/10.18632/oncotarget.6743.>

36. Lu B, Yu Y, Xing XL, Liu RY. miR-183/TMSB4Y, a new potential signaling axis, involving in the progression of laryngeal cancer via modulating cell adhesion. J Recept Signal Transduct Res. 2020:1-8. <https://doi.org/10.1080/10799893.2020.1863987.>

37. Nafian Dehkordi S, Khani F, Hassani SN, Baharvand H, Soleimanpour-Lichaei HR, Salekdeh GH. The Contribution of Y Chromosome Gen es to Spontaneous Differentiation of Human Embryonic Stem Cells into Embryoid Bodies In Vitro. Cell J. 2021;23(1):40-50. <https://doi.org/10.22074/cellj.2021.7145.>

38. Taguchi A, Taylor AD, Rodriguez J, Celiktaş M, Liu H, Ma X, Zhang Q, Wong CH, Chin A, Girard L, et al. A search for novel cancer/testis antigens in lung cancer identifies VCX/Y genes, expanding the repertoire of potential immunotherapeutic targets. Cancer Res. 2014;74(17):4694-705. <https://doi.org/10.1158/0008-5472.CAN-13-3725.>

39. Lahn BT, Page DC. A human sex-chromosomal gene family expressed in male germ cells and encoding variably charged proteins. Hum Mol Genet. 2000;9(2):311-9. <https://doi.org/10.1093/hmg/9.2.311.>

40. Gong Y, Wang L, Chippada-Venkata U, Dai X, Oh WK, Zhu J. Constructing Bayesian networks by integrating gene expression and copy number data identifies NLGN4Y as a novel regulator of prostate cancer progression. Oncotarget. 2016;7(42):68688-68707. <https://doi.org/10.18632/oncotarget.11925.>

41. Ross JL, Tartaglia N, Merry DE, Dalva M, Zinn AR. Behavioral phenotypes in males with XYY and possible role of increased NLGN4Y expression in autism features. Genes Brain Behav. 2015;14(2):137-44. <https://doi.org/10.1111/gbb.12200.>

42. Nguyen TA, Lehr AW, Roche KW. Neuroligins and Neurodevelopmental Disorders: X-Linked Genetics. Front Synaptic Neurosci. 2020;12:33. <https://doi.org/10.3389/fnsyn.2020.00033.>

43. Dong C, Liu Y, Lyu TJ, Beldar S, Lamb KN, Tempel W, Li Y, Li Z, James LI, Qin S, et al. Structural Basis for the Binding Selectivity of Human CDY Chromodomains. Cell Chem Biol. 2020;27(7):827-838.e7. <https://doi.org/10.1016/j.chembiol.2020.05.007.>

44. Stahl PJ, Mielnik AN, Barbieri CE, Schlegel PN, Paduch DA. Deletion or underexpression of the Y-chromosome genes CDY2 and HSFY is associated with maturation arrest in American men with nonobstructive azoospermia. Asian J Androl. 2012;14(5):676-82. <https://doi.org/10.1038/aja.2012.55.>

45. Navarro-Costa P, Plancha CE, Gonçalves J. Genetic dissection of the AZF regions of the human Y chromosome: thriller or filler for male (in)fertility? J Biomed Biotechnol. 2010;2010:936569. <https://doi.org/10.1155/2010/936569.>

46. Liu Y, Liu S, Yuan S, Yu H, Zhang Y, Yang X, Xie G, Chen Z, Li W, Xu B, et al. Chromodomain protein CDYL is required for transmission/restoration of repressive histone marks. J Mol Cell Biol. 2017;9(3):178-194. <https://doi.org/10.1093/jmcb/mjx013.>

47. Kinoshita K, Shinka T, Sato Y, Kurahashi H, Kowa H, Chen G, Umeno M, Toida K, Kiyokage E, Nakano T, et al. Expression analysis of a mouse orthologue of HSFY, a candidate for the azoospermic factor on the human Y chromosome. J Med Invest. 2006;53(1-2):117-22. <https://doi.org/10.2152/jmi.53.117.>

48. Vogt PH, Bender U, Deibel B, Kiesewetter F, Zimmer J, Strowitzki T. Human AZFb deletions cause distinct testicular pathologies depending on their extensions in Yq11 and the Y haplogroup: new cases and review of literature. Cell Biosci. 2021;11(1):60. <https://doi.org/10.1186/s13578-021-00551-2.>

49. Vogt P, Keil R, Kirsch S. The AZF-function of the human Y chromosome during spermatogenesis. Chromosomes today. London: Capman & Hall; 1993. pp . 227–239. <https://doi.org/10.1007/978-94-011-1510-0_18>.

50. Jangravi Z, Tabar MS, Mirzaei M, Parsamatin P, Vakilian H, Alikhani M, Shabani M, Haynes PA, Goodchild AK, Gourabi H, et al. Two Splice Variants of Y Chromosome-Located Lysine-Specific Demethylase 5D Have Distinct Function in Prostate Cancer Cell Line (DU-145). J Proteome Res. 2015;14(9):3492-502. <https://doi.org/10.1021/acs.jproteome.5b00333.>

51. Meyfour A, Pahlavan S, Ansari H, Baharvand H, Salekdeh GH. Down-Regulation of a Male-Specific H3K4 Demethylase, KDM5D, Impairs Cardiomyocyte Differentiation. J Proteome Res. 2019;18(12):4277-4282. <https://doi.org/10.1021/acs.jproteome.9b00395.>

52. Plch J, Hrabeta J, Eckschlager T. KDM5 demethylases and their role in cancer cell chemoresistance. Int J Cancer. 2019;144(2):221-231. <https://doi.org/10.1021/acs.jproteome.9b00395>.

53. Shen X, Hu K, Cheng G, Xu L, Chen Z, Du P, Zhuang Z. KDM5D inhibit epithelial-mesenchymal transition of gastric cancer through demethylation in the promoter of Cul4A in male. J Cell Biochem. 2019;120(8):12247-12258. https://doi.org/10.1002/jcb.27308.

54. Liu M, Gao N. KDM5D inhibits the transcriptional activation of FKBP4 by suppressing the expression of E2F1 in colorectal cancer in males. Biochem Pharmacol. 2021;194:114814. https://doi.org/10.1016/j.bcp.2021.114814.

55. Tian Y, Stamova B, Jickling GC, Xu H, Liu D, Ander BP, Bushnell C, Zhan X, Turner RJ, Davis RR, et al. Y chromosome gene expression in the blood of male patients with ischemic stroke compared with male controls. Gend Med. 2012;9(2):68-75.e3. https://doi.org/10.1016/j.genm.2012.01.005.

56. Yang H, Li Q, Zhang L, Zhu M, Niu J, Xue F, Yang L, Qu Q, Lao Y, Ding Z, et al. LncPRYP4-3 serves as a novel diagnostic biomarker for dissecting subtypes of metabolic associated fatty liver disease by targeting RPS4Y2. Clin Exp Med. 2020;20(4):587-600. https://doi.org/10.1007/s10238-020-00636-1.

57. Lopes AM, Miguel RN, Sargent CA, Ellis PJ, Amorim A, Affara NA. The human RPS4 paralogue on Yq11.223 encodes a structurally conserved ribosomal protein and is preferentially expressed during spermatogenesis. BMC Mol Biol. 2010;11:33. https://doi.org/10.1186/1471-2199-11-33.

58. Kido T, Tabatabai ZL, Chen X, Lau YC. Potential dual functional roles of the Y-linked RBMY in hepatocarcinogenesis. Cancer Sci. 2020;111(8):2987-2999. https://doi.org/10.1111/cas.14506.

59. Alikhani M, Sharifi Tabar M, Mirshahvaladi S, Kheimeh A, Sadighi Gilani MA, Sabbaghian M. Expression analysis of RNA-binding motif gene on Y chromosome (RBMY) protein isoforms in testis tissue and a testicular germ cell cancer-derived cell line (NT2). Iran Biomed J. 2013;17(2):54-61. <https://doi.org/10.6091/ibj.1148.2013.>

60. Chua HH, Tsuei DJ, Lee PH, Jeng YM, Lu J, Wu JF, Su DS, Chen YH, Chien CS, Kao PC, Lee CN, Hu RH, Ni YH, Chang MH. RBMY, a novel inhibitor of glycogen synthase kinase 3β, increases tumor stemness and predicts poor prognosis of hepatocellular carcinoma. Hepatology. 2015 Nov;62(5):1480-96. https://doi.org/10.1002/hep.27996.

61. Chai NN, Salido EC, Yen PH. Multiple functional copies of the RBM gene family, a spermatogenesis candidate on the human Y chromosome. Genomics. 1997 Oct 15;45(2):355-61. https://doi.org/10.1006/geno.1997.4944.

62. Elliott DJ, Ma K, Kerr SM, Thakrar R, Speed R, Chandley AC, Cooke H. An RBM homologue maps to the mouse Y chromosome and is expressed in germ cells. Hum Mol Genet. 1996;5(7):869-74. https://doi.org/10.1093/hmg/5.7.869.

63. Tsuei DJ, Hsu HC, Lee PH, Jeng YM, Pu YS, Chen CN, Lee YC, Chou WC, Chang CJ, Ni YH, et al. RBMY, a male germ cell-specific RNA-binding protein, activated in human liver cancers and transforms rodent fibroblasts. Oncogene. 2004;23(34):5815-22. https://doi.org/10.1038/sj.onc.1207773.

64. Prosser J, Inglis JD, Condie A, Ma K, Kerr S, Thakrar R, Taylor K, Cameron JM, Cooke HJ. Degeneracy in human multicopy RBM (YRRM), a candidate spermatogenesis gene. Mamm Genome. 1996;7(11):835-42. https://doi.org/10.1007/s003359900246.

65. Ma K, Inglis JD, Sharkey A, Bickmore WA, Hill RE, Prosser EJ, Speed RM, Thomson EJ, Jobling M, Taylor K, et al. A Y chromosome gene family with RNA-binding protein homology: candidates for the azoospermia factor AZF controlling human spermatogenesis. Cell. 1993;75(7):1287-95. https://doi.org/10.1016/0092-8674(93)90616-x.

66. Stouffs K, Lissens W, Van Landuyt L, Tournaye H, Van Steirteghem A, Liebaers I. Characterization of the genomic organization, localization and expression of four PRY genes (PRY1, PRY2, PRY3 and PRY4). Mol Hum Reprod. 2001;7(7):603-10. https://doi.org/10.1093/molehr/7.7.603.

67. Choudhary V, Schneiter R. Pathogen-Related Yeast (PRY) proteins and members of the CAP superfamily are secreted sterol-binding proteins. Proc Natl Acad Sci U S A. 2012;109(42):16882-7. https://doi.org/10.1073/pnas.1209086109.

68. Stouffs K, Lissens W, Verheyen G, Van Landuyt L, Goossens A, Tournaye H, Van Steirteghem A, Liebaers I. Expression pattern of the Y-linked PRY gene suggests a function in apoptosis but not in spermatogenesis. Mol Hum Reprod. 2004;10(1):15-21. https://doi.org/10.1093/molehr/gah010.

69. Nakahori Y, Kobayashi K, Komaki R, Matsushita I, Nakagome Y. A locus of the candidate gene family for azoospermia factor (YRRM2) is polymorphic with a null allele in Japanese males. Hum Mol Genet. 1994;3(9):1709. https://doi.org/10.1093/hmg/3.9.1709.

70. Tse JY, Wong EY, Cheung AN, O WS, Tam PC, Yeung WS. Specific expression of VCY2 in human male germ cells and its involvement in the pathogenesis of male infertility. Biol Reprod. 2003;69(3):746-51. https://doi.org/10.1095/biolreprod.103.015792.

71. Wong EY, Tse JY, Yao KM, Lui VC, Tam PC, Yeung WS. Identification and characterization of human VCY2-interacting protein: VCY2IP-1, a microtubule-associated protein-like protein. Biol Reprod. 2004;70(3):775-84. https://doi.org/10.1095/biolreprod.103.018531.

72. Zhang Y, Li M, Xiao F, Teng R, Zhang C, Lan A, Gu K, Li J, Wang D, Li H, et al. Impact of partial DAZ1/2 deletion and partial DAZ3/4 deletion on male infertility. Gene. 2015;571(1):9-16. https://doi.org/10.1016/j.gene.2015.07.083.

73. Fu XF, Cheng SF, Wang LQ, Yin S, De Felici M, Shen W. DAZ Family Proteins, Key Players for Germ Cell Development. Int J Biol Sci. 2015;11(10):1226-35. https://doi.org/10.7150/ijbs.11536.

74. Reynolds N, Cooke HJ. Role of the DAZ genes in male fertility. Reprod Biomed Online. 2005;10(1):72-80. https://doi.org/10.1016/s1472-6483(10)60806-1.

75. Ghorbel M, Baklouti-Gargouri S, Keskes R, Chakroun N, Sellami A, Fakhfakh F, Ammar-Keskes L. gr/gr-DAZ2-DAZ4-CDY1b deletion is a high-risk factor for male infertility in Tunisian population. Gene. 2016;592(1):29-35. https://doi.org/10.1016/j.gene.2016.07.050.

76. Kleiman SE, Yogev L, Hauser R, Botchan A, Bar-Shira Maymon B, Schreiber L, Paz G, Yavetz H. Members of the CDY family have different expression patterns: CDY1 transcripts have the best correlation with complete spermatogenesis. Hum Genet. 2003;113(6):486-92. https://doi.org/10.1007/s00439-003-0990-9.

77. Kleiman SE, Lagziel A, Yogev L, Botchan A, Paz G, Yavetz H. Expression of CDY1 may identify complete spermatogenesis. Fertil Steril. 2001;75(1):166-73. https://doi.org/10.1016/s0015-0282(00)01639-3.

78. Heydarian N, Favaedi R, Sadighi Gilani MA, Shahhoseini M. Expression level of chromodomain Y (CDY): potential marker for prediction of sperm recovery in non-obstructive azoospermia. Int J Reprod Biomed. 2016;14(6):383-8. PMID: 27525321.

79. Ghorbel M, Baklouti-Gargouri S, Keskes R, Chakroun N, Sellami A, Fakhfakh F, Ammar-Keskes L. Combined deletion of DAZ2 and DAZ4 copies of Y chromosome DAZ gene is associated with male infertility in Tunisian men. Gene. 2014;547(2):191-4. https://doi.org/10.1016/j.gene.2014.05.061.

80. Fernandes S, Paracchini S, Meyer LH, Floridia G, Tyler-Smith C, Vogt PH. A large AZFc deletion removes DAZ3/DAZ4 and nearby genes from men in Y haplogroup N. Am J Hum Genet. 2004;74(1):180-7. https://doi.org/10.1086/381132.
